# Intracranial recordings reveal high-frequency activity in the human temporal-parietal cortex supporting non-literal language processing

**DOI:** 10.3389/fnins.2023.1304031

**Published:** 2024-01-08

**Authors:** Shweta Soni, Jacqueline Overton, Julia W. Y. Kam, Penny Pexman, Akshay Prabhu, Nicholas Garza, Ignacio Saez, Fady Girgis

**Affiliations:** ^1^Department of Clinical Neurosciences, University of Calgary, Calgary, AB, Canada; ^2^Department of Neuroscience and Psychiatry, Icahn School of Medicine at Mount Sinai, New York, NY, United States; ^3^Department of Psychology, University of Calgary, Calgary, AB, Canada; ^4^Hotchkiss Brain Institute, University of Calgary, Calgary, AB, Canada; ^5^Department of Neurological Surgery, University of California, Davis, Davis, CA, United States; ^6^Department of Neuroscience, Neurosurgery and Neurology, Icahn School of Medicine at Mount Sinai, New York, NY, United States

**Keywords:** non-literal language processing, electrophysiology, intracranial EEG, neural signal processing, social cognition, neuroimaging, human cognitive neuroscience

## Abstract

**Objective:**

Non-literal expressions such as sarcasm, metaphor and simile refer to words and sentences that convey meanings or intentions that are different and more abstract than literal expressions. Neuroimaging studies have shown activations in a variety of frontal, parietal and temporal brain regions implicated in non-literal language processing. However, neurophysiological correlates of these brain areas underlying non-literal processing remain underexplored.

**Methods:**

To address this, we investigated patterns of intracranial EEG activity during non-literal processing by leveraging a unique patient population. Seven neurosurgical patients with invasive electrophysiological monitoring of superficial brain activity were recruited. Intracranial neural responses were recorded over the temporal-parietal junction (TPJ) and its surrounding areas while patients performed a language task. Participants listened to vignettes that ended with non-literal or literal statements and were then asked related questions to which they responded verbally.

**Results:**

We found differential neurophysiological activity during the processing of non-literal statements as compared to literal statements, especially in low-Gamma (30–70 Hz) and delta (1–4 Hz) bands. In addition, we found that neural responses related to non-literal processing in the high-gamma band (>70 Hz) were significantly more prominent at TPJ electrodes as compared to non-TPJ (i.e., control) electrodes in most subjects. Moreover, in half of patients, high-gamma activity related to non-literal processing was accompanied by delta-band modulation.

**Conclusion:**

These results suggest that both low- and high-frequency electrophysiological activities in the temporal-parietal junction play a crucial role during non-literal language processing in the human brain. The current investigation, utilizing better spatial and temporal resolution of human intracranial electrocorticography, provides a unique opportunity to gain insights into the localized brain dynamics of the TPJ during the processing of non-literal language expressions.

## 1 Introduction

Non-literal figures of speech such as sarcasm and metaphor convey meanings or intentions that are different from their literal expression (Gibbs and Colston, 2012). Because these types of statements are ubiquitous in social communication (Billow, 1981; Gibbs, 2000; Hancock, 2004; Whalen et al., 2009; Burgers et al., 2012; Biddle et al., 2020) and disruptions in processing these types of expressions are implicated in neurodevelopmental disorders (Happé, 1993; Dennis et al., 2001; Mo et al., 2008), it is crucial to understand the neural mechanisms that underlie the processing of these figures of speech.

The interpretation of non-literal language requires an understanding of the context of communication (Searle, 1979; Grice, 1989) as well as the general intention of the speaker (Katz and Pexman, 1997). Therefore, understanding non-literal meanings requires the utilization and conceptual blending of different information that may be simultaneously accessible during language comprehension (Fauconnier and Turner, 2008) which makes the whole process cognitively more engaging and demanding as compared to literal interpretation (Levorato and Cacciari, 2002; Citron et al., 2019). Moreover, the listener requires access to the speaker’s mental states and intents about the utterance to determine meanings that are not literal (Channon et al., 2005; Spotorno et al., 2012). Therefore, non-literal comprehension involves an element of theory of mind, which is defined as the ability to attribute mental states to oneself and others (Premack and Woodruff, 1978). For example, autistic children with impaired theory of mind processing are unable to comprehend complex non-literal statements as compared to neurotypical children with intact theory of mind processing (Happé, 1993; Sullivan et al., 1995).

From a neuroscientific standpoint, because of the relation between the processing of non-literal statements and those involving theory of mind, there is an overlap in brain regions associated with the two constructs. Both theory of mind and non-literal processing incorporate and share various functional networks such as the frontoparietal control network (Cole et al., 2013), the default mode network (Buckner et al., 2008; Andrews-Hanna et al., 2010), and the ventral attention network (Dosenbach et al., 2007), as well as areas implicated in working memory and inhibition (Bohrn et al., 2012; Rapp et al., 2012; Yang, 2014; Reyes-Aguilar et al., 2018). This idea that brain regions enabling theory of mind processes are engaged in comprehending non-literal meanings is reinforced by imaging studies contrasting non-literal and literal language processing (Eviatar and Just, 2006; Wang et al., 2006; Wakusawa et al., 2007; Rapp et al., 2010; Shibata et al., 2010; Prat et al., 2012; Spotorno et al., 2012; Uchiyama et al., 2012; Schneider et al., 2015; Baptista et al., 2018; Filik et al., 2019) and their meta-analyses (Bohrn et al., 2012; Rapp et al., 2012). Recently, Hauptman et al. (2023) suggested that non-literal comprehension is co-supported by mechanisms that process literal meaning and those that facilitate broad social inference such as theory of mind (Hauptman et al., 2023). Therefore, it would be reasonable to expect enhanced BOLD activation during fMRI tasks looking at non-literal comprehension in brain regions that are part of the ToM network, namely the temporal-parietal junction (TPJ), the brain area most often associated with theory of mind (Saxe and Kanwisher, 2003; Leslie et al., 2004; Aichhorn et al., 2009; Spreng and Grady, 2010; Schurz et al., 2014). Because non-literal communication calls upon theory of mind skills (Happé, 1993), these imaging studies serve as compelling evidence for the link between theory of mind and non-literal processing.

In this vein, fMRI studies have found a direct relationship between the TPJ and non-literal processing (Prat et al., 2012; Spotorno et al., 2012). For example, reading phrases that were contextually non-literal (e.g., “*This campaign has really been a hit.*” in the context of a disappointing campaign) activated bilateral TPJ when compared to reading phrases that were contextually literal (e.g., “*This campaign has really been a hit.*” in the context of an excellent campaign) (Spotorno et al., 2012). This finding was corroborated by other studies exploring neural substrates involved in non-literal processing that demonstrated BOLD activation in both hemispheres in regions that anatomically are part of the TPJ. These regions included, the middle and superior temporal gyri, and the inferior parietal cortex (Lee and Dapretto, 2006; Stringaris et al., 2006; Rapp et al., 2007; Boulenger et al., 2009; Mashal et al., 2009; Bosco et al., 2017).

In addition to fMRI studies, scalp EEG studies have employed spectral analysis of electrophysiological responses to gain insights into how brain oscillations are modulated at different frequencies during non-literal comprehension (van den Brink et al., 2012; Spotorno et al., 2013; Sun et al., 2022; Champagne-Lavau et al., 2023; Hubbard et al., 2023). For example, understanding sentences conveying nonliteral meaning elicited an increase in gamma, alpha and delta power as compared to sentences that conveyed literal meaning, suggesting the engagement of different mechanisms such as integration of multiple information and executive functioning while understanding nonliteral sentences (Spotorno et al., 2013). Moreover, the semantic decoding of non-literal expressions (i.e., metaphors) requires more attention and efforts as compared to literal expressions, as indexed by enhanced synchronization in delta and theta band during different time windows throughout widespread brain regions (Sun et al., 2022).

Non-literal language interpretation is associated with increased TPJ activity in fMRI studies and general electrical oscillatory perturbations on scalp EEG studies. While these techniques are non-invasive and well-validated, they can be limited by low spatial resolution (scalp EEG), low temporal (fMRI) resolution, and poor signal-to-noise ratio for individual trials. Intracranial EEG can help mitigate these drawbacks due to its excellent temporal and spatial precision as well as high signal-to-noise ratio, which can better elucidate the role of low and high-frequency oscillatory activity in the TPJ during non-literal processing. Therefore, we examined intracranial EEG (iEEG) from neurosurgical patients undergoing awake brain operations in the region of the TPJ during a task investigating non-literal versus literal language comprehension. Specifically, we examined whether comprehending non-literal statements, such as sarcasm, metaphor, and simile, gives rise to distinct patterns of brain oscillatory activity in the TPJ as compared to comprehending literal statements. This novel methodology offers a more comprehensive depiction of the neurobiology of non-literal language processing. Given the link between the TPJ and non-literal processing, we hypothesized that non-literal processing would elicit a stronger response in low (1–30 Hz) and high (>30 Hz) frequency bands as compared to literal processing in the TPJ and that greater low and high frequency activity would be observed at TPJ electrodes as compared to non-TPJ electrodes.

## 2 Materials and methods

### 2.1 Participants

Seven neurosurgical patients (age in years: 58.14 ± 13.71) undergoing awake craniotomies for clinical purposes were recruited to participate in the study. All participants consented to the study according to the guidelines of the Declaration of Helsinki that was approved by the institutional ethics review board of the University of California, Davis (approval number: 1201780-12). Participants reported no history of other illnesses and did not show any signs of impairment in executive functioning and language skills. Patients were chosen if they met the following specific criteria: They must be able to participate in a cognitive task, the required operation must expose the temporal-parietal junction cortex (TPJ), and this exposed cortex must be normal, meaning that it possesses normal signal characteristics on standard MRI sequences and it appears normal on direct visualization during surgery. As per routine clinical practice in awake brain surgery, surface strip electrodes were used to record after-discharge potentials during eloquent cortex mapping to minimize the risk of seizures and enhance the safety of the mapping procedure. Once clinical mapping was complete, and if the patient was comfortable, fully alert, cooperative, and able to participate in a cognitive task, the strip electrodes were repositioned over the TPJ, and the cognitive task was administered for research purposes. The data for two patients were not usable because the patients did not tolerate testing: one patient became nauseated at the beginning of the task and could not continue, and a second opted out of the study after a few trials. Data from another patient were excluded because of noisy electrophysiologic recordings that resulted in most trials being discarded during analysis. Therefore, data from three patients were excluded from the analyses. The remainder of this section pertains to the remaining four patients whose data were included in the analyses (see Table 1 for patients’ demographic details).

**Table 1:** Participant demographics and testing details.

Sub#	Age	G	Handed-ness	Diagnosis	Surgery side	# of TPJ	# of non-TPJ	# of vignettes completed	Accuracy
S01	65	F	Right	Tumor	Right	8	5	5	85%
S02	61	M	Right	Tumor	Left	3	3	5	63.2%
S03	44	F	Right	Tumor	Left	3	3	6	100%
S04	76	M	Right	Tumor	Left	3	3	5	57.9%

### 2.2 Surgery and electrode localization

Patients underwent a craniotomy to expose the brain for mapping and subsequent tumor resection. Prior to resection, patients were implanted with 1 to 4 strips (6 to 8 channel) of platinum-iridium electrodes, each with a 3 mm exposed recording area and 10 mm center to center inter-contact spacing (Ad-Tech Medical, Racine, WI). Intra-operative photos were taken once the strips were in position, and these were then used to map exact electrode locations on a 3-dimensional MRI surface reconstruction for each patient. The anatomical location of each electrode was determined by the neurosurgeon (e.g., TPJ, primary sensory cortex, superior temporal gyrus, etc.), and locations projected onto a 3-D brain rendering generated from an anatomical atlas within an online neuroimaging software suite (Freesurfer, Charlestown, MA) using MATLAB (Mathworks, Natick, MA). Importantly, anatomical coverage across patients was based on clinical requirements. All patients except for one underwent left sided operations as per the standard clinical strategy of carrying out speech mapping in the left hemisphere.

**Figure 1:**
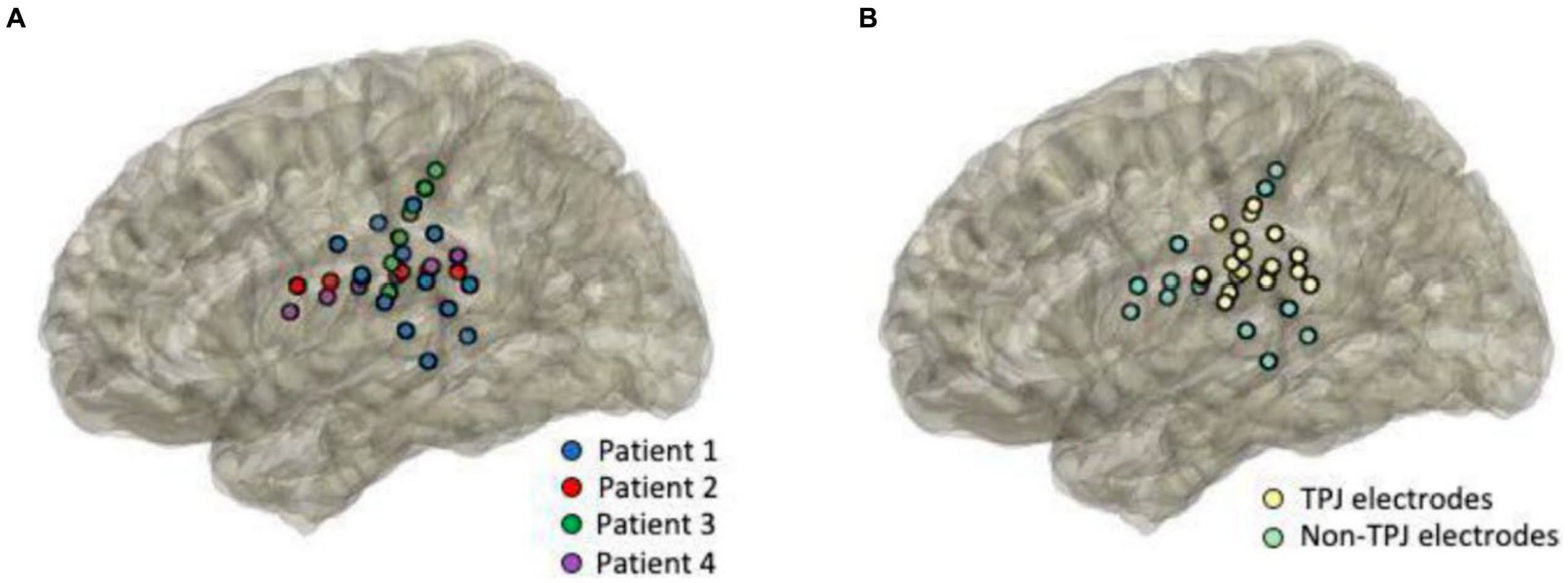
Anatomical coverage. Intracranial cortical EEG responses were recorded while patients performed the language task. Electrodes were positioned directly over the surgically exposed cortex, centered around the temporal-parietal junction (TPJ). Images represent anatomical reconstruction of the electrode projections onto a template brain. Electrodes are color-coded by patients [**(A)**, *n* = 4] and by anatomical classification [**(B)**, TPJ in yellow and non-TPJ in light green].

Electrode strips were placed directly onto the cortical surface and positioned over the TPJ area for experimental testing (Figure 1). Electrodes that were placed within the anatomical borders of the TPJ were classified as TPJ electrodes. Electrodes outside of the TPJ were placed over a variety of adjacent areas, including motor and auditory cortices, and were categorized as non-TPJ (control) electrodes (see Figure 1A for individual patient electrode placement and Figure 1B for anatomical classification of electrodes). All patients had at least 3 contacts in the TPJ area (mean number of TPJ contacts = 4.25 ± 2.5, range: 3–8) as well as in non-TPJ areas (mean number of non-TPJ contacts = 3.5 ± 1, range: 3–5), but the number of channels included in the analysis was lower (mean TPJ = 2.75 ± 1.5, mean non-TPJ = 2 ± 0) due to our bipolar derivation analytical strategy (TPJ/non-TPJ: 5/2 for S01, 2/2 for S02, S03, and S04). Bipolar referencing is considered advantageous for analyzing iEEG signals as it emphasizes activity with high local specificity by removing signal artifacts shared by nearby electrodes and widely dispersed neural response components (Mercier et al., 2022). Electrodes were excluded from analyses if there was abnormal tissue at that site.

### 2.3 Language task

We adopted a simple mentalization task (Happé, 1994; Stone et al., 1998; Jolliffe and Baron-Cohen, 1999; Gregory et al., 2002) in which participants make inferences about the mental states and intentions of others based on statements made in different situations. An audio testing format was chosen over a visual or computer-based format due to the limitations associated with the surgical conditions. Participants were lying supine on the operating table with their heads fixed in a clamp and turned approximately 70 degrees. In addition, there were sterile drapes impeding parts of their visual field. Therefore, looking at a computer screen or reading a paragraph of text was too onerous for the participants, making a listening task with verbal responses the most feasible option.

**Figure 2:**
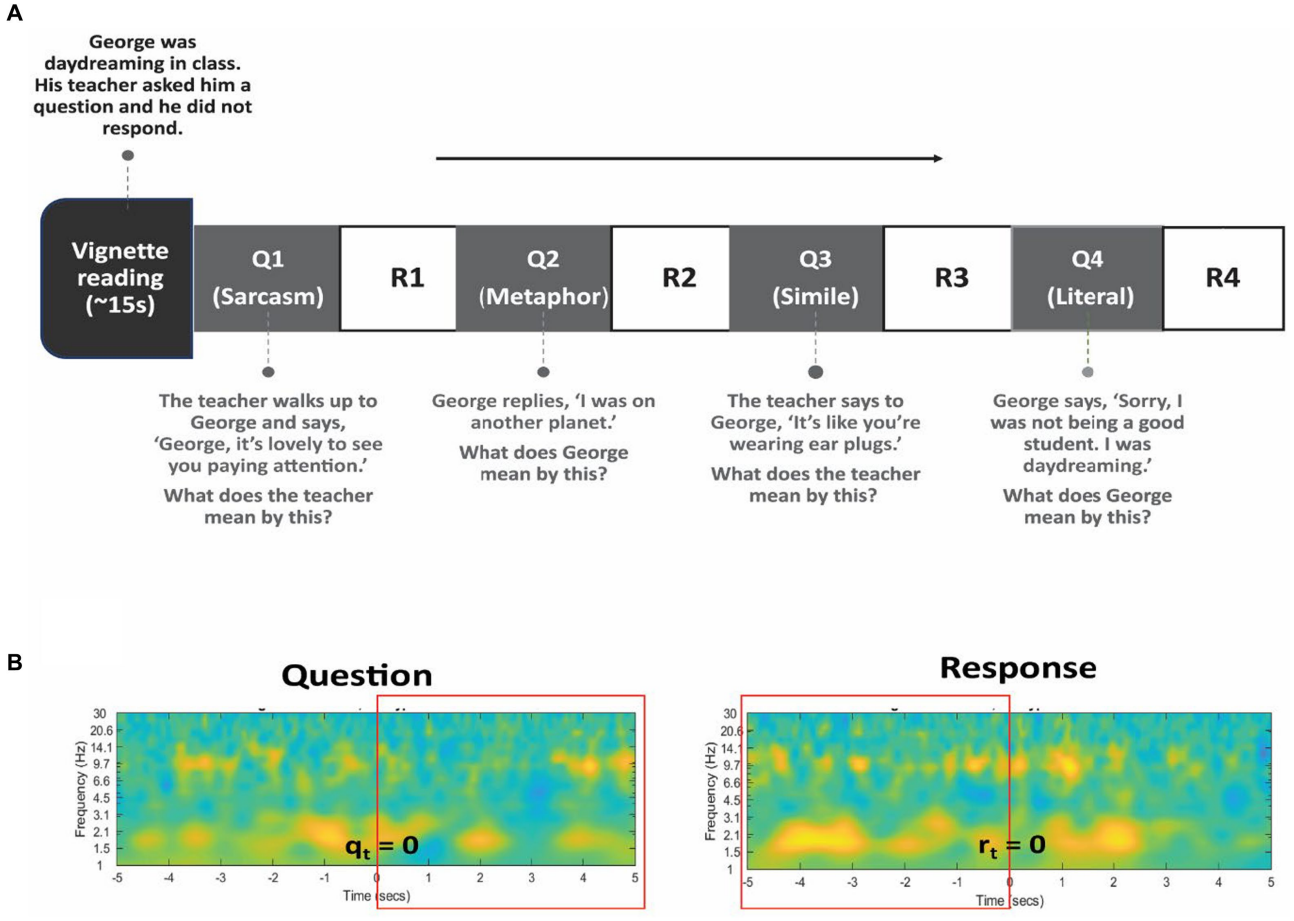
Language task design. **(A)** The subject listened to several vignettes read aloud describing brief interpersonal situations. Following each vignette, 4 questions were posed to assess comprehension of non-literal (Q1, Q2, and Q3) and literal (Q4) statements. Verbal responses (R1 to R4) were recorded. An example vignette and questions are shown. **(B)** The rectangular box in red color represents the time-window chosen for Question (left) and for Response (right) period in all analyses. Specifically, the five seconds following the presentation of the question (q_t_ = 0) and the five seconds preceding the verbal response (r_t_ = 0) is chosen to capture the relevant cognitive processes.

Participants were read a series of vignettes which presented brief interpersonal situations (see Supplementary material). These were stories adapted from initial psychological work on theory of mind (Happé, 1994) and expanded to involve various non-literal figures of speech. Following each vignette, four questions were asked that required different forms of mentalization and assessed the participant’s ability to comprehend non-literal and literal expressions in speech (Figure 2A). The first three questions of each vignette required the interpretation and identification of non-literal statements, including sarcasm, metaphor, and simile, while the fourth question involved interpreting a literal statement. Participants responded verbally, and audio was recorded using an external microphone and timestamped to EEG signals. Testing time was limited to approximately 10 min due to surgical constraints, during which time patients were asked to listen to and respond to the four questions to a maximum of six vignettes. The behavioral performance was indexed by the percentage accuracy of the completed questions. In addition to the percentage accuracy, we measured reaction times after each question.

### 2.4 iEEG recording and analyses

iEEG was recorded during the language task in all available channels and timestamped with events in the task to allow parsing of neuronal activity in relation to task epochs. Number of channels varied across participants between 6 and 13 (see Table 1). Channels were amplified × 10,000, analog filtered (0.01–1,000 Hz) with a 4,096 Hz digitization rate (except in one case where a digitization rate of 2,048 Hz was used). Electrophysiological data were imported to MATLAB for preprocessing and analyses with custom scripts. Channels were visually examined, and channels with low signal-to-noise ratio were identified and excluded from subsequent analyses (i.e., electromagnetic noise from hospital equipment, amplifier saturation, poor contact with cortical surface). Signals were down sampled to 1,024 Hz, high-pass filtered above 1.0 Hz and lowpass filtered below 200 Hz with symmetrical (phase true) finite impulse response filters (~35 dB/octave roll-off), and notch filtered to remove line noise (60 Hz and harmonics), using order 8 Butterworth filters. Pairs of adjacent channels were subtracted to produce bipolar re-referenced signals (e.g., given 6 original channels, channels 2 and 5 were selected as reference for channels 1, 3 and 4, 6 respectively, yielding 4 resultant bipolar signals). Periods with excessive noise (e.g., due to patient movement) or epileptiform activity were identified using a custom-written algorithm followed by visual inspection, marked, and excluded from analysis (i.e., timepoints around artifacts were set to NaNs). Because our data consisted of a small number of trials of relatively long duration, we did not reject entire trials due to noise or artifact as is commonly done, but rather excluded noisy periods from statistical analysis and maintained the remainder of the trials.

To obtain an initial comprehensive picture of task-related changes in neural responses, we generated power spectra and event-related spectrograms. For that, we estimated time-frequency representations (TFRs) using complex Morlet wavelet convolution (Cohen, 2019) for each trial excluding artifactual periods as described above. We applied wavelet transform separately in a range of 1 to 30 Hz (in 24 log-spaced steps) and in a range of 30 to 200 Hz (in 30 log-spaced steps) frequencies based on appropriate number of wavelet cycles for both frequency ranges. We examined both low and high frequency bands for several reasons. First, task-related activations during cognitive tasks may occur in several frequency bands (Lakatos et al., 2005). Second, past studies have reported greater synchronization in several frequency bands during non-literal comprehension (Spotorno et al., 2013; Sun et al., 2022; Champagne-Lavau et al., 2023). Finally, the high-frequency broadband activity (>70 Hz) is considered a marker of cortical activation (Parvizi and Kastner, 2018) and related to both neuronal population spiking and BOLD activation (Logothetis et al., 2001; Engell et al., 2012; Jacques et al., 2016; Leszczyński et al., 2023). Given fMRI studies have reported increased activation during non-literal comprehension in the TPJ, this may be manifested in the high-frequency broadband activity in the iEEG signal.

Each trial was separated into a question and response period, with the onset of the audio presentation of the vignettes marking the onset of the question (q_t_ = 0) and the onset of participants’ verbal response as the onset of the response period (r_t_ = 0). Spectral power for each period was calculated and baseline corrected in reference to a 4 s time-window prior to the first question. The first three questions comprising the non-literal condition were combined for analysis. So, we averaged spectral power for all non-literal conditions and literal conditions separately across different time-windows for the question (0 to +5 s) and response (−5 to 0 s) period to capture the relevant cognitive processes (Figure 2B). Specifically, the five seconds following the presentation of the question and the five seconds preceding the verbal response presumably engaged the processing of non-literal and literal statements and assessing underlying intentions or meanings. The mean spectral power for non-literal and literal conditions was also separately averaged across TPJ electrodes and non-TPJ electrodes. We carried out two main analyses described below.

To address our first hypothesis, we delineated the neurophysiological response to non-literal and literal conditions by comparing TFRs for non-literal conditions to TFRs for the literal condition (non-literal vs. literal contrast) using the cluster-based permutation method (between-condition comparison). These TFRs have two dimensions: temporal (time-points) and spectral (frequencies). The mean spectral power of each sample— (time, frequency)-pair in a TFR— in the non-literal condition was contrasted to the mean spectral power of the equivalent sample in the literal condition by using the Kolmogorov–Smirnov (KS) statistic and summing the KS-statistic value across connected components or ‘cluster’ (adjacent (time, frequency)-pairs) that exceeded the KS value at an alpha level of 0.05. This led to a large number of statistical comparisons as the difference between non-literal and literal conditions is evaluated at all samples in the time-frequency plane. To correct for the multiple comparisons, we performed a Monte Carlo simulation by shuffling the trial assignment for each pair of TFRs (i.e., randomly assigning trials to the non-literal and literal conditions), recalculating the KS contrast and sum of KS statistic across suprathreshold (alpha <0.05) clusters, and repeating this process 1,000 times. Significant or suprathreshold clusters were identified by taking the 99th percentile of the resulting distribution of 1,000 KS-cluster sizes (cluster alpha <0.01) and applying this threshold to the original unshuffled KS-statistic TFR contrasts that resulted into cluster-corrected TFRs. This procedure was repeated using each participant’s TFR data and the resulting cluster corrected TFRs were separately obtained for both low (1–30 Hz) and high (30–200 Hz) frequencies in question and response periods at TPJ and non-TPJ electrodes.

To summarize the neural activity from these cluster-corrected TFRs, we calculated the ‘proportion of active timepoints’ in discrete frequency bands: delta (1 to 4 Hz), theta (4 to 8 Hz), alpha (8 to 13 Hz), beta (13 to 30 Hz), low-gamma (LG: 30 to 70 Hz) and high-gamma (HG: 70 to 200 Hz). To carry out this analysis, we binary coded each time point in the time-frequency plane as active (i.e., if cluster corrected significant activity exists in any frequency at that time point) or inactive (i.e., if no significant clusters emerged at that time point). We then summed the number of active time points (based on suprathreshold clusters) in a particular frequency band within the event of interest (0 to +5 s for question and −5 to 0 s for response) and divided this by the total number of timepoints in that frequency band in the time-frequency plane. Thus, the proportion of active timepoints gives an estimate of the engagement of different frequency bands across the task at both types of electrodes.

To address our second hypothesis, we assessed the differences in non-literal processing-related neural responses between TPJ and non-TPJ electrodes in the above-mentioned frequency bands (between-electrode comparison). Though measuring the proportion of active timepoints shows differences in neural responses between TPJ and non-TPJ electrodes, between-electrode comparison of this metric acquired from cluster-corrected TFRs of only four participants would not provide enough statistical power. Therefore, we took a different approach and first identified timepoints for suprathreshold clusters from the cluster corrected TFR (representing non-literal versus literal contrast) within each frequency band. These clusters indicate significantly more neural activity while processing non-literal statements than literal statements. We then extracted spectral power that corresponds to the timepoints of these suprathreshold clusters from the non-literal TFRs during the question and response periods in each trial and averaged across the period of interest within each frequency band. We referred to it as ‘power of active timepoints’, representing neural activity in different frequency bands during non-literal processing. This approach not only allowed us to execute trial-balanced comparisons between TPJ and non-TPJ regions but also provided us with sufficient statistical power using more data points (instead of one data point per participant).

### 2.5 Statistical analysis

All statistical analyses were performed using customized scripts in MATLAB (2022b). Descriptive data are presented as mean ± standard deviation unless otherwise mentioned. We used non-parametric tests because our data is not normally distributed. Comparisons between experimental conditions or electrode types were examined using two-tailed Wilcoxon sign rank test (non-parametric equivalent for matched-samples comparisons) with significance level set at 0.05.

## 3 Results

Our analyses were based on four patients undergoing awake craniotomy exposing normal temporal-parietal junction (TPJ). They completed at least 4 of 6 language task vignettes (5 ± 0.81 vignettes completed on average). Behavioral performance on the completed questions was high, with mean accuracy of 76.51 (±16.95) across all conditions. Mean accuracy varied slightly across conditions: non-literal questions overall: 75 ± 16.58 (sarcasm: 65 ± 35.7; metaphor: 90 ± 10; simile: 70 ± 17.32) and literal questions: 81.25 ± 20.73. This indicates patients understood and performed the task adequately. Participants took slightly longer while responding to non-literal questions: overall: 7.07 ± 0.93 (sarcasm: 5.16 ± 1.31; metaphor: 8.09 ± 1.40; simile: 7.96 ± 1.19) than literal questions: 6.29 ± 1.38. However, the overall reaction time for non-literal questions was not significantly different from the reaction time for literal questions (*p* = 0.25, Wilcoxon sign-rank test). In addition, we did not find a significant relation between the percentage of accuracy and reaction time for any type of question (Pearson correlation coefficient and value of *p* for sarcasm: 0.10 and 0.90; metaphor: −0.51 and 0.45, simile: −0.55 and 0.41, literal: 0.50 and 0.46). This signifies that participant’s ability to understand and answer correctly did not depend upon the speed they responded with. All the non-literal questions were pooled together in neurophysiological analyses (see Methods).

**Figure 3:**
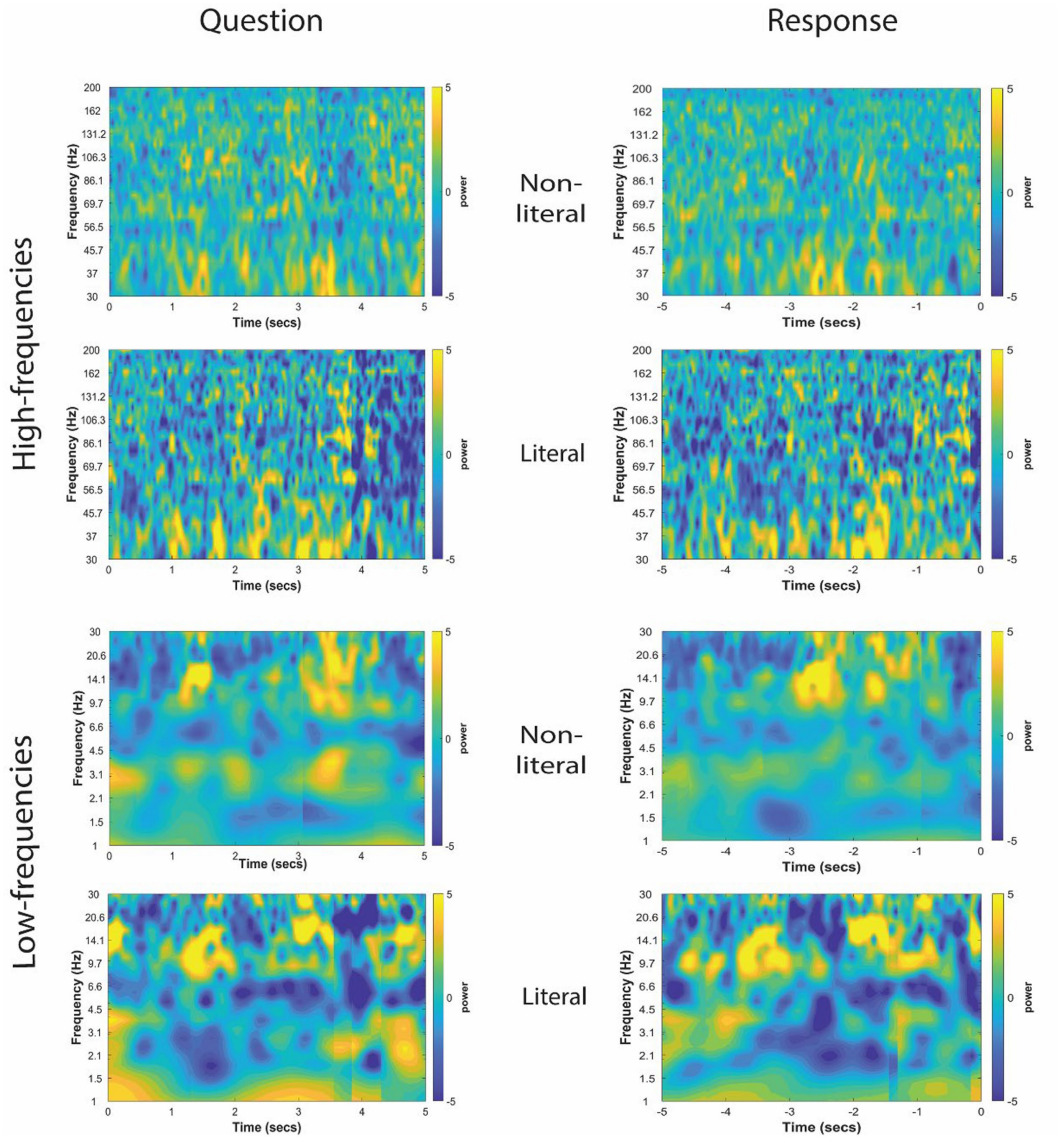
Time-frequency representation of intracranial EEG activity at TPJ electrodes during question and response periods in an example participant. Y-axis and X-axis represent frequency in Hertz and time in seconds, respectively. For the Question (left column), *t* = 0 indicates the start time of the question being read by the experimenter, and for the Response (right column) *t* = 0 indicates the start time of the participant’s verbal response. Time-frequency representations were averaged for both non-literal and literal conditions showing differential power modulations in both high (30–200 Hz, top panel) and low (1–30 Hz, bottom panel) frequencies.

**Figure 4:**
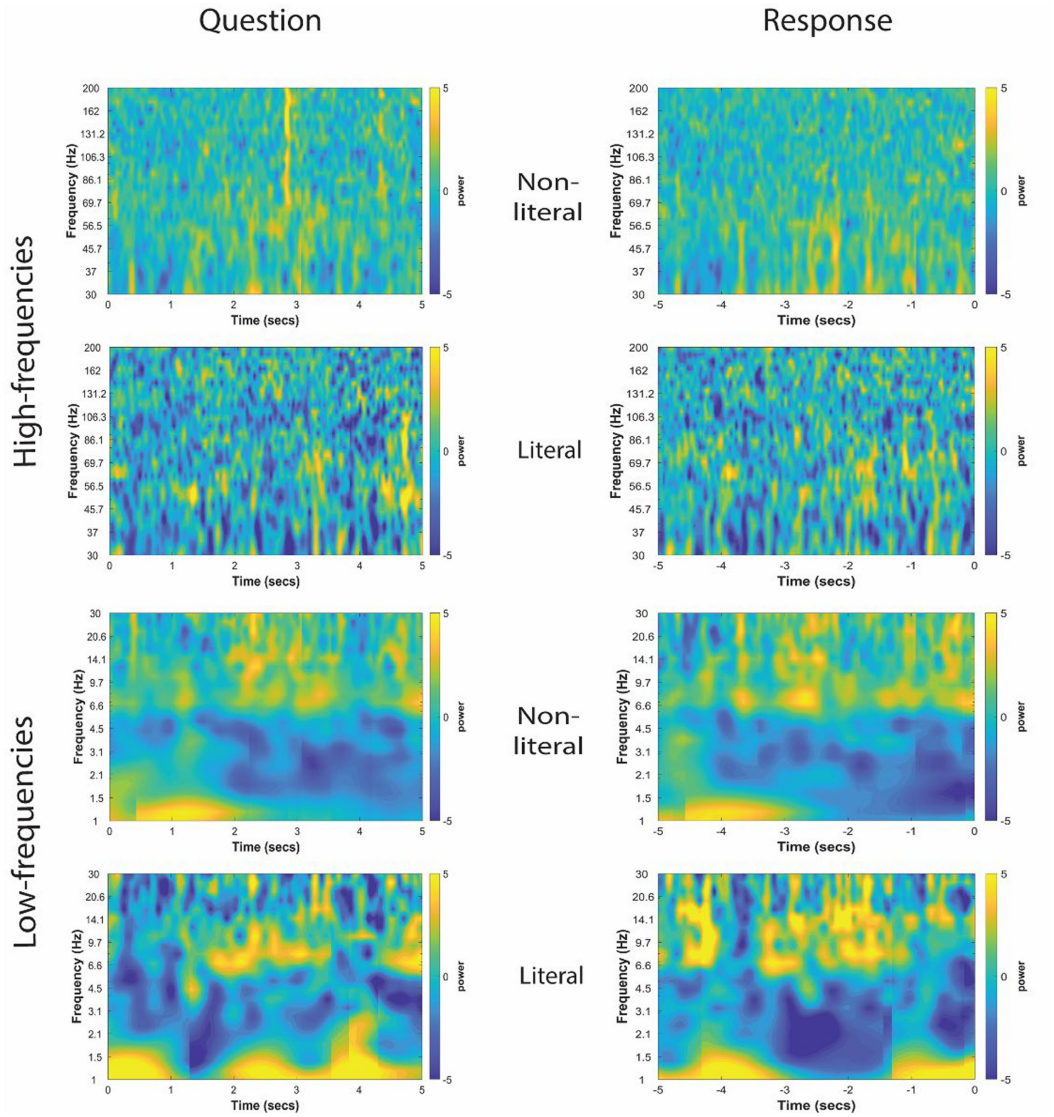
Time-frequency representation of intracranial EEG activity at non-TPJ (i.e., control electrodes) during question and response in an example participant. *Y*-axis and *X*-axis represent frequency in Hertz and time in seconds, respectively, where *t* = 0 indicates the beginning of the Question reading by the experimenter (left column) or of the verbal Response made by the patient (right column). Time-frequency representations were averaged for both non-literal and literal conditions showing differential power modulations in both high- (30–200 Hz, top panel) and low- (1–30 Hz, bottom panel) frequencies.

The TFRs for non-literal and literal conditions from an example patient at TPJ and non-TPJ electrodes during the question and response periods are shown in Figures 3, 4 respectively. In this study, we (1) analyzed non-literal versus literal contrast using a cluster-based permutation approach to examine neural activity specific to non-literal processing at TPJ and non-TPJ electrodes and (2) compared neural activity during non-literal processing between both types of electrodes. The results for both comparisons are described in Sections 3.1 and 3.2, respectively.

### 3.1 Neural activity differs in non-literal and literal processing mainly in low-gamma and delta bands regardless of electrode type

**Figure 5:**
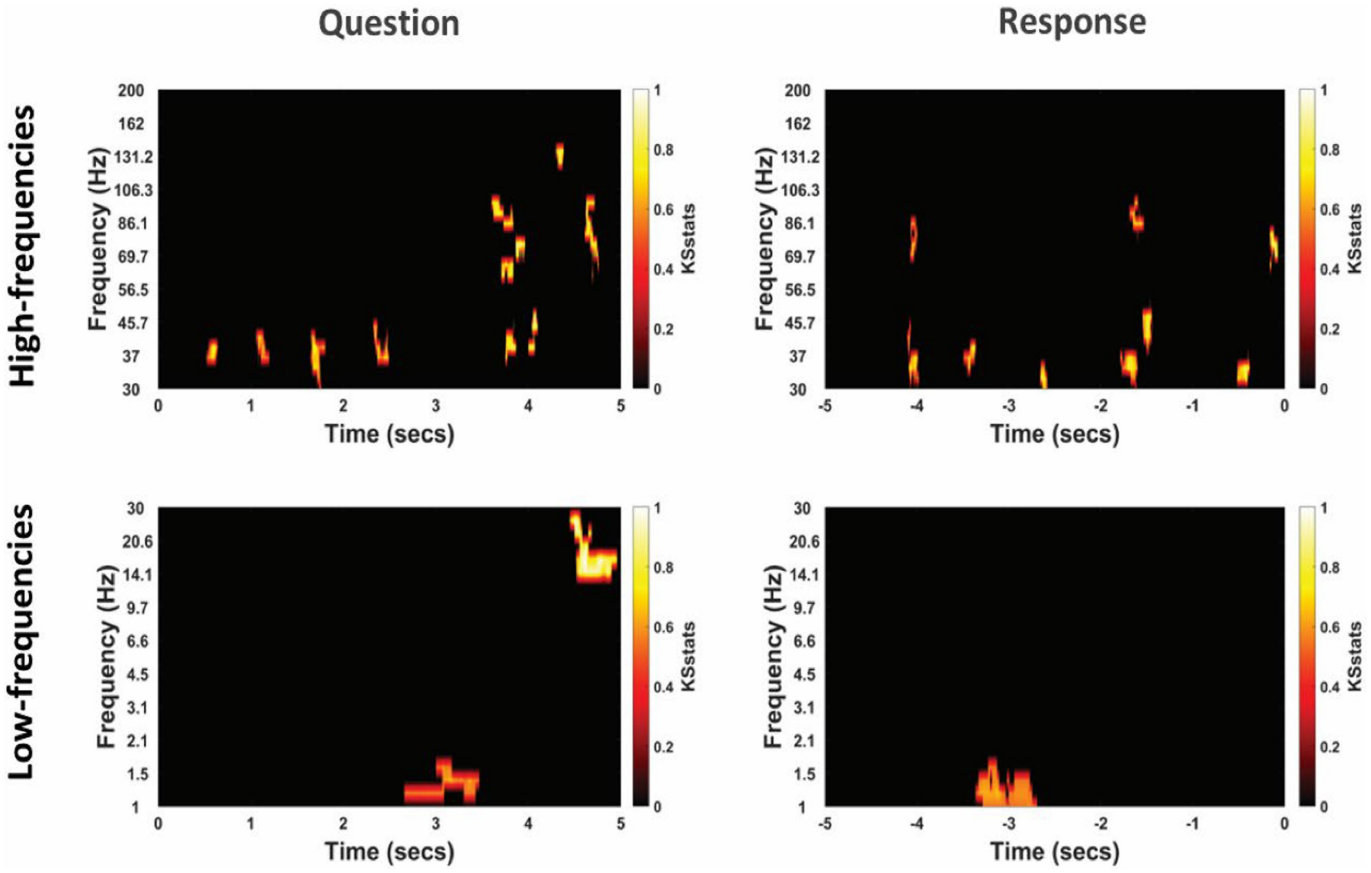
Differential power modulation between non-literal and literal conditions at TPJ electrodes for an example participant. Statistical maps show significant differences in power between non-literal and literal conditions after corrected by cluster-based permutation at *p* = 0.01. Analyses were performed separately for Question (left column) and Response (right column) epochs in both high-frequency (top panel) as well as low-frequency (bottom panel) bands. The *t* = 0 at the x-axis of these maps indicates the time when the Question was started to be read by the experimenter or when the verbal Response began by the patient. Color intensity of clusters represent KS-statistics resulting from comparing power across all questions/responses and across all TPJ electrodes for each condition (non-literal/literal).

**Figure 6:**
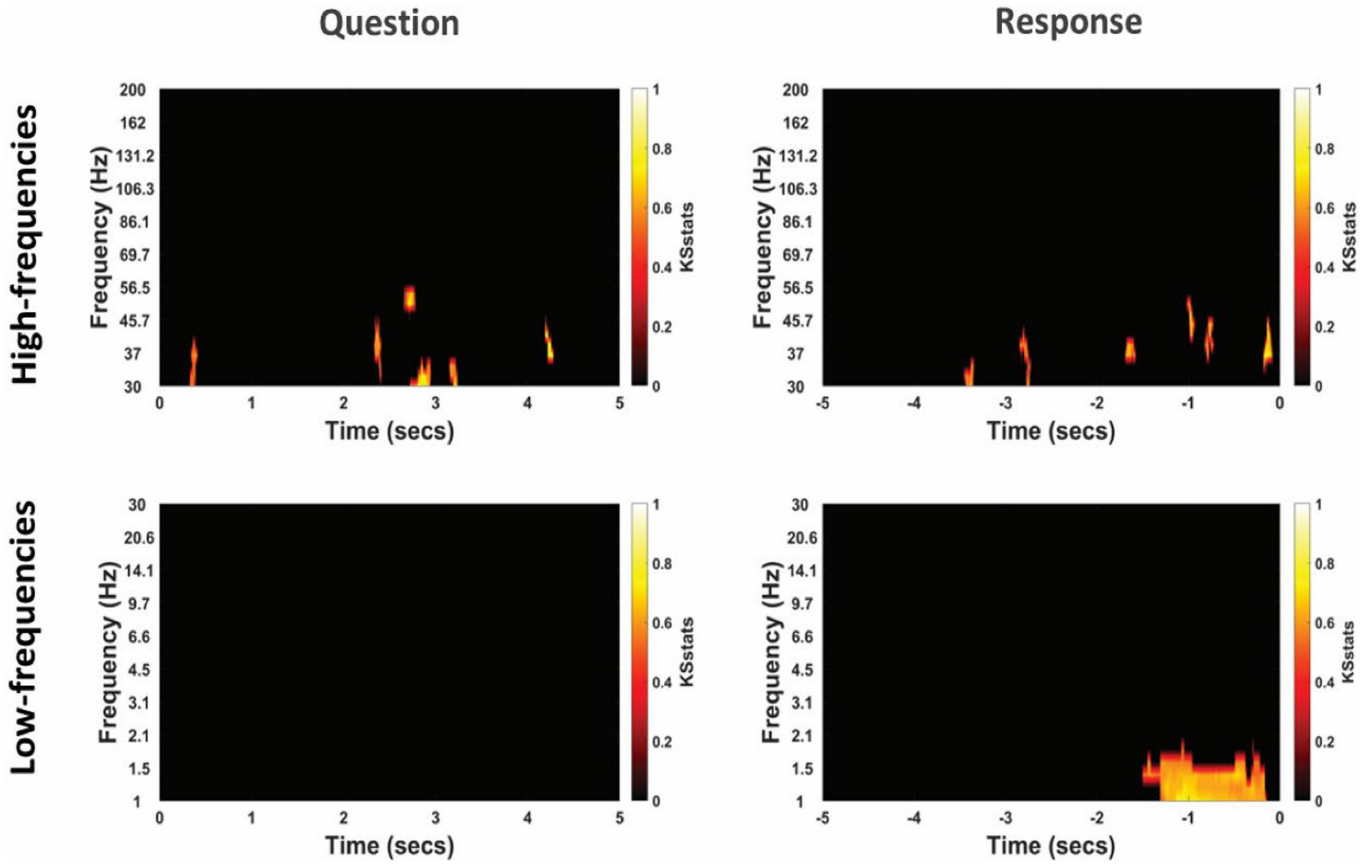
Differential power modulation between non-literal and literal conditions at non-TPJ or control electrodes for an example participant. Statistical maps show significant differences in power between non-literal and literal conditions after corrected by cluster-based permutation at *p* = 0.01. Analyses were performed separately for Question (left column) and Response (right column) epochs in both high-frequency (top panel) as well as low-frequency (bottom panel) bands. The *t* = 0 at the x-axis of these maps indicates the time when the Question was started to be read by the experimenter or when the verbal Response began by the participant. Color intensity of clusters represent KS-statistics resulting from comparing power across all questions/responses and across all non-TPJ electrodes for each condition (non-literal/literal).

The cluster-corrected spectral activity from the non-literal versus literal contrast in TPJ and non-TPJ electrodes are shown for an example patient in Figures 5, 6 respectively. Clusters showing significantly increased activity in the non-literal condition as compared to literal condition were not sustained in both question and response periods, but rather appeared in discrete temporal patches. Also, the pattern of increased activity varied across patients.

To quantify the involvement of different frequency bands, we summarized the spectral activity related to non-literal versus literal processing as the proportion of active timepoints in each frequency band (see Methods). The results revealed relatively consistent activity in the low-gamma band (30–70 Hz) band at TPJ electrodes, which was present in most patients in our dataset during both question (3 out of 4 participants, min = 0% (no activity at all), max = 2.73% active timepoints) and response (4 out of 4 participants, min = 0.27%, max = 6.30%) periods. This pattern was also similar at non-TPJ electrodes as 3 out of 4 participants showed activity in the low-gamma band in both question (min = 0%, max = 2.15%) and response (min = 0%, max = 1.88%) periods. This proportion of active timepoints was not different between these two periods in any frequency band (*p* > 0.25 at both TPJ non-TPJ electrodes).

Interestingly, there was also activity in the high-gamma band (70–200 Hz) and the delta frequency band (1–4 Hz), but of a less consistent nature, appearing mainly at TPJ electrodes (see Supplementary Table S1). In the high-gamma band, the activity was found only in 2 of 4 patients (1.20 and 1.12%) in the question period and in 3 of 4 patients (1.18, 0.18 and 0.62%) during the response period, respectively. Noticeably, delta activity was consistently found in all patients during the question period (min = 1.37%, max = 6.35%) but was visible only in 2 out of 4 patients (4.30 and 3.33%) in the response period. The TPJ activity in other frequency bands was negligible (overall mean active points in question and response = 1.56 and 1.41%) for theta [4–8 Hz], 0.07 and 0.68% for alpha [8–12 Hz] and 0.96 and 0.02% for beta [12–30 Hz], as well as non-consistent (in question period, 2/4, 1/4, and 1/4 participants for theta, alpha and beta respectively; in response period, 1/4 participants for all three bands). Thus, low-gamma activity might be the marker of neural activity that differs in non-literal and literal processing, which was accompanied by delta-band and high-gamma activity in more than half of the cases at TPJ electrodes. The proportion of active timepoints was not different between question and response periods (*p* > 0.05).

### 3.2 High-gamma power increases at TPJ to a greater extent than at non-TPJ electrodes

**Figure 7:**
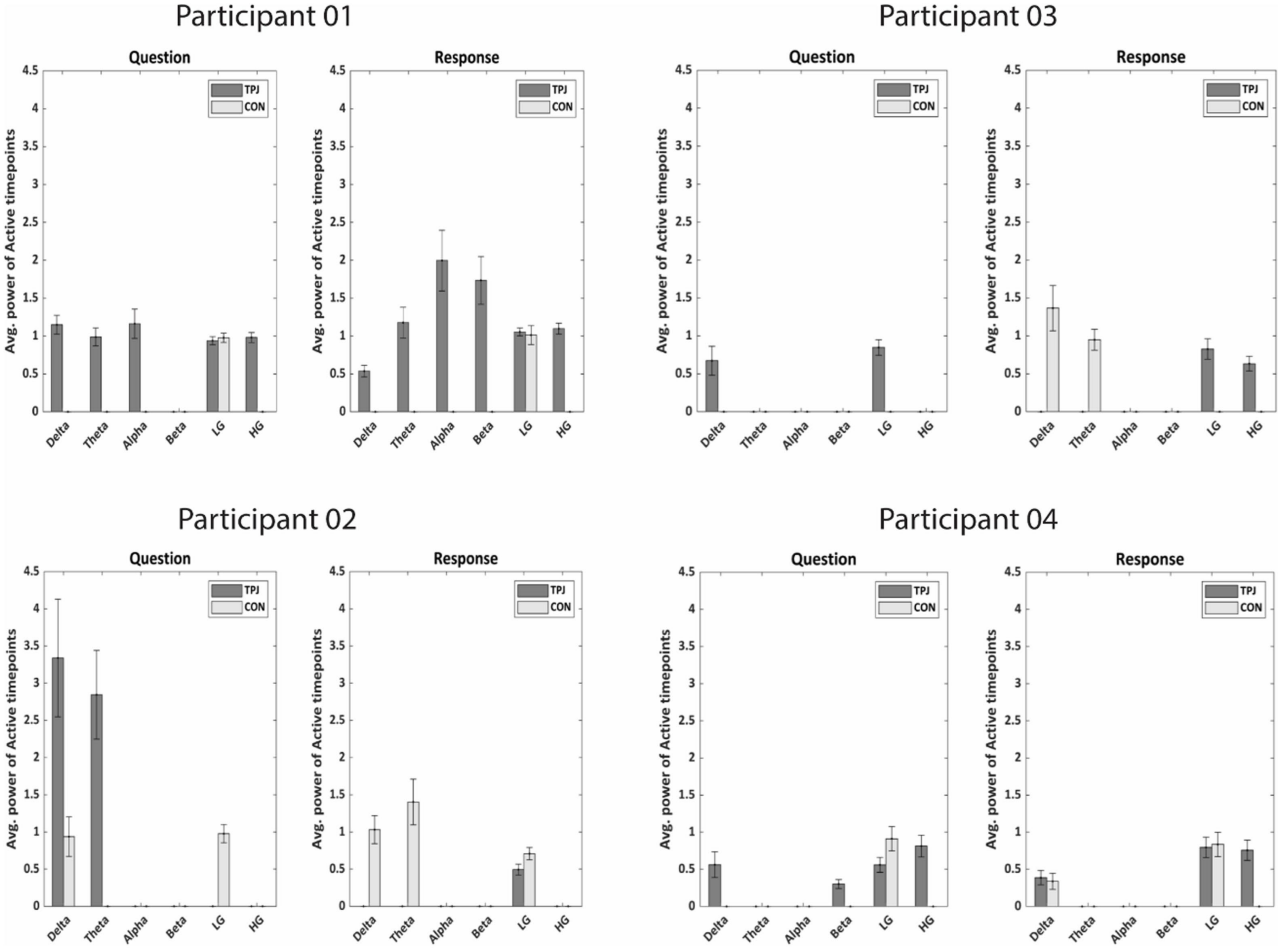
Average power of active timepoints (in μV^2^/Hz) specific to non-literal processing for both TPJ and non-TPJ (CON = control) electrodes in all frequency bands for each individual participant. In each participant, the power of significantly active clusters from non-literal versus literal contrast were averaged across all Questions/Responses and across electrodes for both TPJ and non-TPJ control electrodes. The averaged powers were then compared in all frequency bands separately. Notably, the active clusters were averaged between −5 to 0 s for Questions and between 0 to 5 s for Responses. The comparison shows significantly more power related to non-literal processing at TPJ electrodes than non-TPJ electrodes (Wilcoxon sign-rank test, *p* = 0.01) for most subjects, especially in delta (1–4 Hz) and high-gamma ranges [LG, lower-gamma (1–30 Hz); HG, high-gamma (70–200 Hz)]. Notably, certain frequency bands do not show bars due to the absence of any significantly active clusters resulting from non-literal versus literal contrast in those frequency bands. This indicates that there were no significant differences between non-literal and literal conditions in those frequency bands.

When comparing whether neural activity related to non-literal processing differs in both types of electrodes, our results demonstrate more power of active timepoints at TPJ sites than non-TPJ sites during non-literal-specific processing across participants, mainly in the delta and high-gamma band (see Figure 7). During the question period all participants showed significantly more power at TPJ than non-TPJ in the delta band (*p* < 0.001, Wilcoxon sign-rank test). Moreover, significantly more high-gamma TPJ-power accompanied with delta activity in 2 out of these 4 participants (*p* < 0.001 and *p* = 0.0002 respectively, Wilcoxon sign-rank test). We found a similar pattern of more high-gamma activity in TPJ electrodes during the response period which was present in 3 of 4 participants (*p* < 0.001, *p* < 0.001 and *p* = 0.002 respectively, Wilcoxon sign-rank test). However, we did not find any robust pattern of more power at TPJ than non-TPJ across participants in any other frequency bands such as theta, alpha, beta and low gamma, as only 1 or 2 out of 4 participants showed more TPJ activity in these bands during both question and response periods. Hence, high-gamma and delta activity seem to be indicators of TPJ-specific activity during non-literal processing.

## 4 Discussion

We investigated patterns of neurophysiological activity involved in interpreting situations that included non-literal expressions in comparison to literal expressions. Importantly, our study is the first, to our knowledge, to directly examine the involvement of the TPJ in processing non-literal stimuli using an iEEG approach. Particularly, we sought to verify if (1) processing non-literal situations elicit different neural activity compared to literal situations at TPJ and non-TPJ sites, and if (2) the TPJ sites show greater activity during non-literal processing than non-TPJ sites (i.e., electrodes placed over motor or auditory areas). In general, our results demonstrate that iEEG activation for the non-literal condition was significantly different from that for the literal condition in low-gamma (30–70 Hz), high-gamma (70–200 Hz) and delta (1–4 Hz) bands at the TPJ. Specifically, we noticed a robust increase in low-gamma activity across the majority of patients during both question (while the patient was listening to a question about the content of vignettes) and response (before the patient responded to the question) periods. We further show that TPJ electrodes exhibit more non-literal processing-specific activation than non-TPJ electrodes, mainly in the high-gamma (70–200 Hz) band. We found no evidence for an activity increase in other low-frequency bands (theta, alpha, beta).

The key strength of the present work is that it employs intracranial EEG to reveal brain oscillatory dynamics during non-literal processing. The use of intracranial electrodes that capture task-relevant activity from only a few millimeters of the cortex provided direct access to ‘local’ signals from highly precise anatomical localizations (Holdgraf et al., 2016). In addition to offering good spatial resolution, intracranial EEG enabled the recording of high-frequency activity (broadband gamma, 30–200 Hz). This would be difficult to achieve using non-invasive approaches where the skull functions as a low-pass filter, attenuating signals above ~40 Hz (Buzsáki et al., 2012). Additionally, investigation of brain activity using time-frequency analysis can reveal subtle differences in brain oscillations that cannot be captured by ERP analyses alone (Cohen, 2014).

We quantified the brain oscillatory activity specific to non-literal processing by conducting a between-condition comparison of time-frequency representations (non-literal and literal contrast) and summarized in canonical frequency bands as proportion of active timepoints. One significant finding of this analysis was that patients robustly showed a higher proportion of active timepoints in the low-gamma band both while assessing the situation (question period) and formulating an answer (response period). This finding suggests that low-gamma activity may support the processing of non-literal situations, which is consistent with earlier research that reported increased power in the low-gamma band associated with non-literal processing that is different from literal processing (Spotorno et al., 2013). Unlike that study which observed increased power in the range of 30–35 Hz, low-gamma activation in our study was broader, between 30–70 Hz. We attribute this difference to task design, stimuli and methodologies used in the two investigations. While Spotorno et al. (2013) employed sentences where the target sentence was presented with a context conveying negative or opposite meaning (ironic condition), our stimuli involved a range of contexts to cue non-literal meanings. Also, the intracranial EEG seems to capture task-related changes in higher frequencies better than the scalp EEG. Broadly, our results are consistent with the reported role of low-gamma in the integration of various information streams in the semantic domain, such as bringing together real-world knowledge and literal meanings (Hagoort et al., 2004; van den Brink et al., 2012).

It is noteworthy that the low-gamma activity specific to non-literal processing was observed in both TPJ and non-TPJ areas. We speculate that increased low-gamma activity during nonliteral versus literal comprehension only reflects mechanisms that enable the unification of semantic and social information. Other mechanisms such as mental inferencing, which is another important aspect of nonliteral understanding and involves inferring others’ mental states and intents, are not fully captured by the low-gamma activity in our investigation. If this were true, only TPJ electrodes—which are critical in facilitating theory of Mind processes (Saxe and Kanwisher, 2003; Young et al., 2010)—would exhibit an increase in low-gamma activity. Future research, however, will be required to disentangle linguistic from mentalizing factors that support non-literal interpretation.

The between-electrode analysis performed on the power of significantly active timepoints revealed stronger high-gamma activation at TPJ electrodes than non-TPJ electrodes in 2 and 3 of 4 patients during question and responses periods, respectively, in our dataset. This suggests that it may be a marker of non-literal processing despite differences in anatomical localization across TPJ sub-regions, which varied across patients. Given that the TPJ has traditionally been implicated in ToM processes (Channon et al., 2005), one likely explanation of this finding is that the high-gamma activity is related to ToM-related mechanisms involved in non-literal comprehension. In non-literal situations, our participants must be assessing characters’ mental states and intentions in addition to literal meanings, based on contextual information given in vignettes (Hauptman et al., 2023). This is reflected in high-gamma oscillations during both question and response periods. The increased TPJ activity in our study is corroborated by previous investigations that found enhanced BOLD activation during non-literal comprehension in brain regions (such as medial prefrontal cortex, TPJ) that support ToM processes (Rapp et al., 2010; Shibata et al., 2010; Prat et al., 2012; Spotorno et al., 2012; Uchiyama et al., 2012; Baptista et al., 2018). Thus, our study provides a bridge between non-invasive imaging studies and localized electrophysiological activity normally not observable in human brains.

We also found non-literal processing-related activity in lower frequency bands, particularly in the delta band (1–4 Hz). For both between-condition as well as between-electrode comparisons, delta band activity was highly increased at the TPJ across all participants in question periods but only in 1 and 2 out of 4 participants in response periods. Overall, this makes delta activation comparatively a less robust but an important marker of non-literal comprehension. Functionally, delta oscillations are related to attentional processes (Knyazev, 2012; Ko et al., 2017), and found to reflect executive functioning while understanding non-literal sentences (Spotorno et al., 2013; Sun et al., 2022). Thus, we cannot rule out differences in the attentional state of patients and its effects on neural activity. However, the lack of a relationship between behavioral performance and proportion of active timepoints in the delta band (Pearson correlation, *p >* 0.05) in our data suggests these are not related (see Supplementary Table S2). Despite this caveat, slow ongoing oscillations may be a good candidate to coordinate neuronal activity in engaged brain areas during continuous (i.e., extended over a long period of time and not stimulus-locked) processes, such as those necessary to understand and assign agency during the question period, which takes at least five seconds in our experimental design. Compared to the temporally precise, local high frequency activity, oscillations in low frequencies are thought to integrate over large spatial regions and long temporal scales (Canolty and Knight, 2010). Given they were observed mainly at TPJ electrodes, these slow oscillations may serve to broadcast relevant ToM-related information needed for interpreting non-literal meaning to other associated brain areas and support interaction between other nodes engaged in non-literal processing.

Interestingly, we did not see consistent activity in the intermediate frequency bands (theta, alpha, beta). We were particularly expecting theta activity (4–8 Hz) based on previous research on non-literal processing (Sun et al., 2022), and due to its association with higher-order cognitive processing in temporal-parietal cortex (Brunetti et al., 2014; Meyer et al., 2015; Wang et al., 2016; Cooper et al., 2017; Bramson et al., 2018). For example, magnetoencephalography recordings during a visual perspective task found a spike in theta power in right posterior temporal-parietal cortex during perspective taking (‘embodying’ another’s viewpoint) but not perspective tracking (merely tracking what another person can see), and this embodied processing could be significantly reduced with transcranial magnetic stimulation to the same area (Wang et al., 2016). We did see greater theta activity at TPJ than non-TPJ sites in question periods in both analyses but only in half of our participants. The lack of consistency in theta activation may be related to the inclusion of non-literal expressions other than metaphors (Sun et al., 2022), or perhaps because our task did not elicit a strong degree of perspective-taking.

One limitation in the present study is that we combined three different types of expressions (sarcasm, metaphor, and simile) under the ‘non-literal’ condition for our analysis. The primary reason to do so was to take our experimental complexity into account. The experimental conditions were difficult given that subjects were awake on the operating room table, and that resulted in limited number of participants and trials per condition. In addition to increasing the number of trials, combining our non-literal expressions allowed us to improve the statistical power of the TPJ versus non-TPJ contrast during non-literal sentence processing. That being said, there are specific differences in terms of the inferencing involved in these expressions, as well as similarities; for instance, they all need extralinguistic information to be interpreted (see Gibbs and Colston (2012) for an overview of standard pragmatic models). The processing of metaphors mainly relies upon analogical reasoning (Willinger et al., 2017) and structural mappings (Gentner and Bowdle, 2008). Like metaphors, similes are comprehended through controlled, comparison processes (Gentner and Bowdle, 2008; Pambuccian and Raney, 2021). Contrarily, the processing of sarcasm uses frame-shifting and conceptual blending operations for semantic reorganization when inconsistencies between incoming information and an initial interpretation are found (Coulson, 2001). Moreover, sarcasm processing also requires social conceptual knowledge (Akimoto et al., 2014) as well as the understanding of the higher-order nature of the speaker’s belief (Pexman and Glenwright, 2007; Gibbs and Colston, 2012). We do not completely disregard the possibility that the interpretation of our current findings may be confounded by the processing variations among different non-literal expressions and hence suggest an important direction for further exploration will be to investigate processing of each nonliteral type separately.

In conclusion, the present work enhances our understanding of the neurophysiological basis of TPJ activation while processing non-literal expressions. Leveraging a unique dataset, invasive intracranial recordings from neurosurgical patients, we demonstrate that low-gamma activity is robustly enhanced while understanding non-literal statements as compared to literal statements, both during listening to a question and contemplating a response. In addition to low-gamma activation, delta activity, which reflects slower oscillatory dynamics, increases strongly but inconsistently. We further show that high-gamma activation related to non-literal processing is enhanced to a greater extent in the TPJ area than non-TPJ areas. Future studies are recommended to investigate the causal role of TPJ in the comprehension of non-literal expressions and how multi-frequency oscillations operate to coordinate neuronal activity across implicated brain regions.

## Data availability statement

The raw data supporting the conclusions of this article will be made available by the authors, without undue reservation.

## Ethics statement

The studies involving humans were approved by the institutional ethics review board and was in accordance with the guidelines of the Declaration of Helsinki. The studies were conducted in accordance with the local legislation and institutional requirements. The participants provided their written informed consent to participate in this study.

## Author contributions

SS: Conceptualization, Formal analysis, Methodology, Software, Visualization, Writing – original draft, Writing – review & editing. JO: Formal analysis, Methodology, Software, Writing – original draft, Writing – review & editing. JK: Conceptualization, Supervision, Writing – review & editing. PP: Conceptualization, Supervision, Writing – review & editing. AP: Investigation, Methodology, Writing – review & editing. NG: Investigation, Methodology, Writing – review & editing. IS: Conceptualization, Supervision, Writing – review & editing. FG: Conceptualization, Investigation, Supervision, Writing – review & editing.
