# Supplementary material for: Intracranial recordings reveal high-frequency activity in the human temporal-parietal cortex supporting non-literal language processing
Source: Front Neurosci. 2024 Jan 8;17:1304031. doi: 10.3389/fnins.2023.1304031 (PMC10800947; doi:10.3389/fnins.2023.1304031)
Supplement: Supplementary file 1 [file Data_Sheet_2.PDF]

Supplementary Materials

Experimental Stimuli

1	George was daydreaming in class. His teacher asked him a question and he did not respond.<br><br>Sarcasm: The teacher walks up to George and says, ‘George, it’s lovely to see you paying attention.’ What does the teacher mean by this?<br>Metaphor: George replies, ‘I was on another planet.’ What does George mean by this?<br>Simile: The teacher says to George, ‘It’s like you’re wearing earplugs.’ What does the teacher mean by this?<br>Literal: George says, ‘Sorry, I was not being a good student. I was daydreaming.’ What does George mean by this?
2	Ken is a very experienced motor mechanic. When he comes home from work his son gives him a toy truck to fix. All that has to be done is tighten a loose screw. Ken takes the truck from his son.<br><br>Sarcasm: He smiles at his wife who has been watching and says, ‘Well, this is quite hard.’ What does Ken mean by this?<br>Metaphor: While fixing the truck he says, ‘Well, this is a piece of cake.’ What does Ken mean by this?<br>Simile: His wife walks in and asks how it is going. Ken says, ‘This is as easy as pie.’ What does Ken mean by this?<br>Literal: His wife says, ‘Well, this looks like a simple problem.’ What does the wife mean by this?
3	Colin has just started university. His father is getting quite annoyed about how little work Colin does. Colin rarely goes to lectures. He is always out at parties. It is 11a.m. on Monday morning and Colin has just gotten out of bed.<br><br>Sarcasm: Colin’s brother says, ‘Dear me, what a busy life you have.’ What does the brother mean by this?<br>Metaphor: Colin’s mum comes in. She has been hanging the washing on the line. She sees Colin still in his pyjamas and says, ‘Dear me, you don’t have much on your plate.’ What does the mum mean by this?<br>Simile: Colin looks up and the mum says, ‘You look like you’re on school holidays.’ What does the mum mean by this?<br>Literal: Colin’s brother is about to leave. He says goodbye. As he leaves, he says to Colin, ‘Dear me, what an easy life you have.’ What does the brother mean by this?
4	A father is playing with his little one-year-old daughter. She is very tiny for her age. To make her laugh, he lifts her high up over his head and twirls her around.<br><br>Sarcasm: He laughs and says, ‘What a heavy weight!’ What does the father mean by this?<br>Metaphor: He twirls her around again and says, ‘You’re a feather.’ What does the father mean by this?<br>Simile: She is twirling and twirling, and the father says, ‘You’re like a balloon.’ What does the father mean by this?

	Literal: He puts his daughter down and says, ‘What a lightweight!’ What does the father mean by this?
5	Mrs. Roberts had been working on her feet all day. She walked so much that her shoes gave her blisters. She finally got home. The first thing she did was take off her uncomfortable shoes and put on her slippers.<br><br>Sarcasm: ‘Ah’ she says, ‘these slippers are so uncomfortable.’ What does Mrs. Roberts mean by this?<br>Metaphor: Mr. Roberts gets home just after Mrs. Roberts. He says hello and asks how she is. Mrs. Roberts says, ‘I’m fine now that I have pillows on my feet.’ What does Mrs. Roberts mean by this?<br>Simile: Mr. Roberts smiles at his wife. Mrs. Roberts talks about how she was running around all day on the hard hospital floor. She says, ‘Oh, but now I feel like I’m walking on cotton wool.’ What does Mrs. Roberts mean by this?<br>Literal: Her husband asks, ‘Why’s that?’ Mrs. Roberts says, ‘Now I’m wearing uggies.’ What does Mrs. Roberts mean by this?
6	It is Greg’s birthday. He will be five. His grandmother is coming over to give him a gift. Greg hopes that it will be a toy truck. When his grandmother arrives, Greg opens the present. It is a jumper, not a toy truck! Greg throws the jumper on the floor and runs out of the room.<br><br>Sarcasm: Greg’s grandmother says to Greg’s mother, ‘Well, he likes it a lot, doesn’t he?’ What does the grandmother mean by this?<br>Metaphor: After Greg ran out, his mum says, ‘Well, he got out of the wrong side of the bed.’ What does the mum mean by this?<br>Simile: Greg’s older sister Jane is also in the room. She says, ‘He’s like a sour puss.’ What does Jane mean by this?<br>Literal: Grandmother, mum and Jane all look at each other. Greg has still not come back. Grandmother says, ‘Well, he doesn’t like it very much, does he?’ What does the grandmother mean by this?
7	Jim and Bill have gone to watch their favorite band play. Unfortunately, they were not able to get in. The tickets were all sold out. However, they might still be able to see something through a window. Jim climbs up onto Bill’s shoulders to try to reach the window. But Jim is huge. Poor Bill nearly collapses under Jim’s weight.<br><br>Sarcasm: Bill says, ‘What a lightweight!’ What does Bill mean by this?<br>Metaphor: After a minute, Bill is still holding Jim up. Bill says, ‘You’re a lump of lead.’ What does Bill mean by this?<br>Simile: Bill finally puts Jim down. Bill looks at Jim and says, ‘You’re like a tonne of bricks.’ What does Bill mean by this?<br>Literal: Bill then says, ‘What a heavy weight!’ What does Bill mean by this?

Supplementary Tables

Supplementary Table 1. Mean (SD) % proportion of active timepoints averaged over subjects in all frequency bands, showing more activation in the low-gamma and delta bands

FREQ. BAND	QUESTION		RESPONSE	
	TPJ	CON	TPJ	CON
Delta	2.881(2.029)	.551 (.954)	1.908 (1.939)	3.034 (3.25)
Theta	1.556 (1.616)	0	1.410 (2.442)	1.382 (1.61)
Alpha	.067 (.116)	0	.683 (1.184)	0
Beta	.958 (1.66)	0	.021 (.037)	0
Low-Gamma (LG)	1.727 (1.065)	1.287 (.789)	2.518 (2.330)	.854 (.708)
High-Gamma (HG)	.579 (.58)	0	.496 (.457)	0

Supplementary Table 2. Pearson correlation between overall behavioral accuracy and proportion of active timepoints

	Delta	Theta	Alpha	Beta	Low-gamma	High-gamma
Question	r = 0.22<br>p = 0.77	r = -0.02<br>p = 0.98	r = 0.29<br>p = 0.69	r = -0.63<br>p = 0.31	r = 0.14<br>p = 0.85	r = -0.27<br>p = 0.71
Response	r = -0.19<br>p = 0.79	r = 0.29<br>p = 0.69	r = 0.29<br>p = 0.69	r = 0.29<br>p = 0.69	r = 0.17<br>p = 0.82	r = 0.09<br>p = 0.91

Supplementary Figures

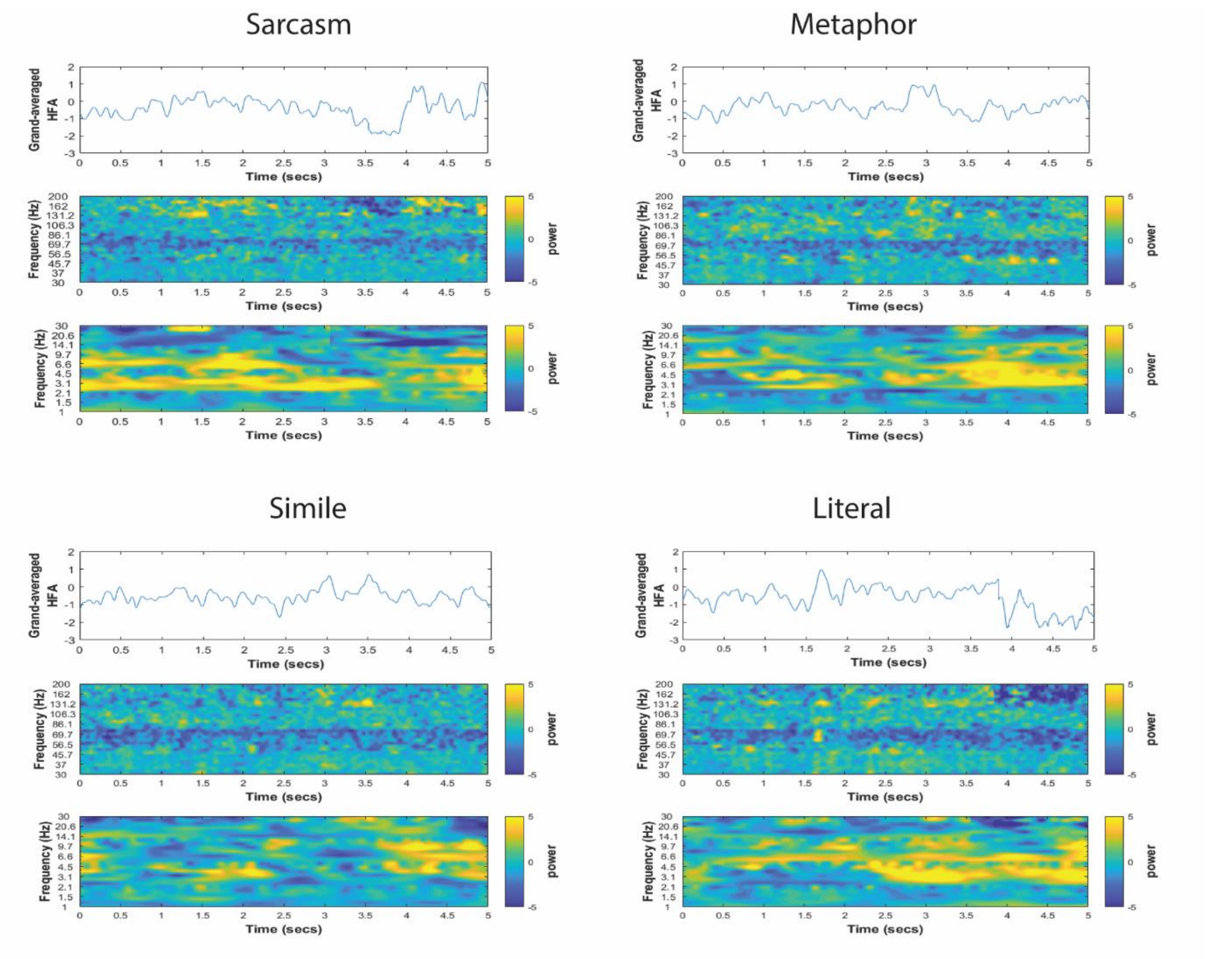

Supplementary Figure 1A. Grand-averaged high-frequency activity (HFA) and grand-averaged time-frequency representations of intracranial EEG activity at TPJ electrodes during question period. For the Question, t=0 indicates the start time of the question being read by the experimenter. For a given condition (sarcasm, metaphor, simile and literal), spectral power was averaged within high-gamma frequency band (70-200 Hz) across all questions and then across all participants at TPJ electrodes to calculate grand-averaged HFA (*top* panel). X-axis and Y-axis represent time in seconds and power of HFA (in μV^2), respectively. For a given condition (sarcasm, metaphor, simile and literal), time-frequency representations were averaged across all questions and then across all participants, showing differential power modulations in both high (30-200 Hz, *middle* panel) and low (1-30 Hz, *bottom* panel) frequencies. X-axis and Y-axis represent time in seconds and frequency (in Hertz).

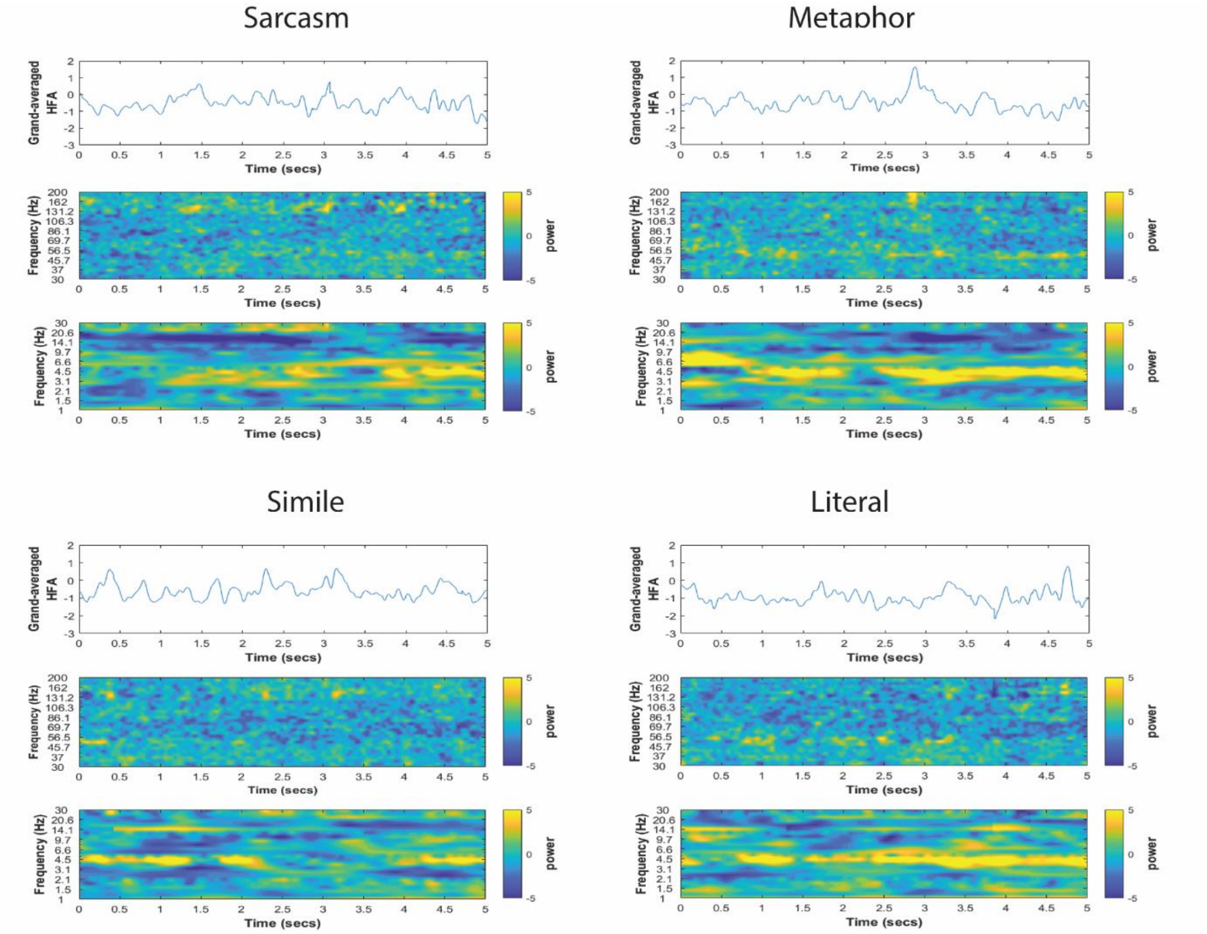

Supplementary Figure 1B. Grand-averaged high-frequency activity (HFA) and grand-averaged time-frequency representations of intracranial EEG activity at non-TPJ electrodes during question period. For the Question, t=0 indicates the start time of the question being read by the experimenter. For a given condition (sarcasm, metaphor, simile and literal), spectral power was averaged within high-gamma frequency band (70-200 Hz) across all questions and then across all participants at non-TPJ electrodes to calculate grand-averaged HFA (*top* panel). X-axis and Y-axis represent time in seconds and power of HFA (in μV^2), respectively. For a given condition (sarcasm, metaphor, simile and literal), time-frequency representations were averaged across all questions and non-TPJ electrodes and then across all participants, showing differential power modulations in both high (30-200 Hz, *middle* panel) and low (1-30 Hz, *bottom* panel) frequencies. X-axis and Y-axis represent time in seconds and frequency (in Hertz).

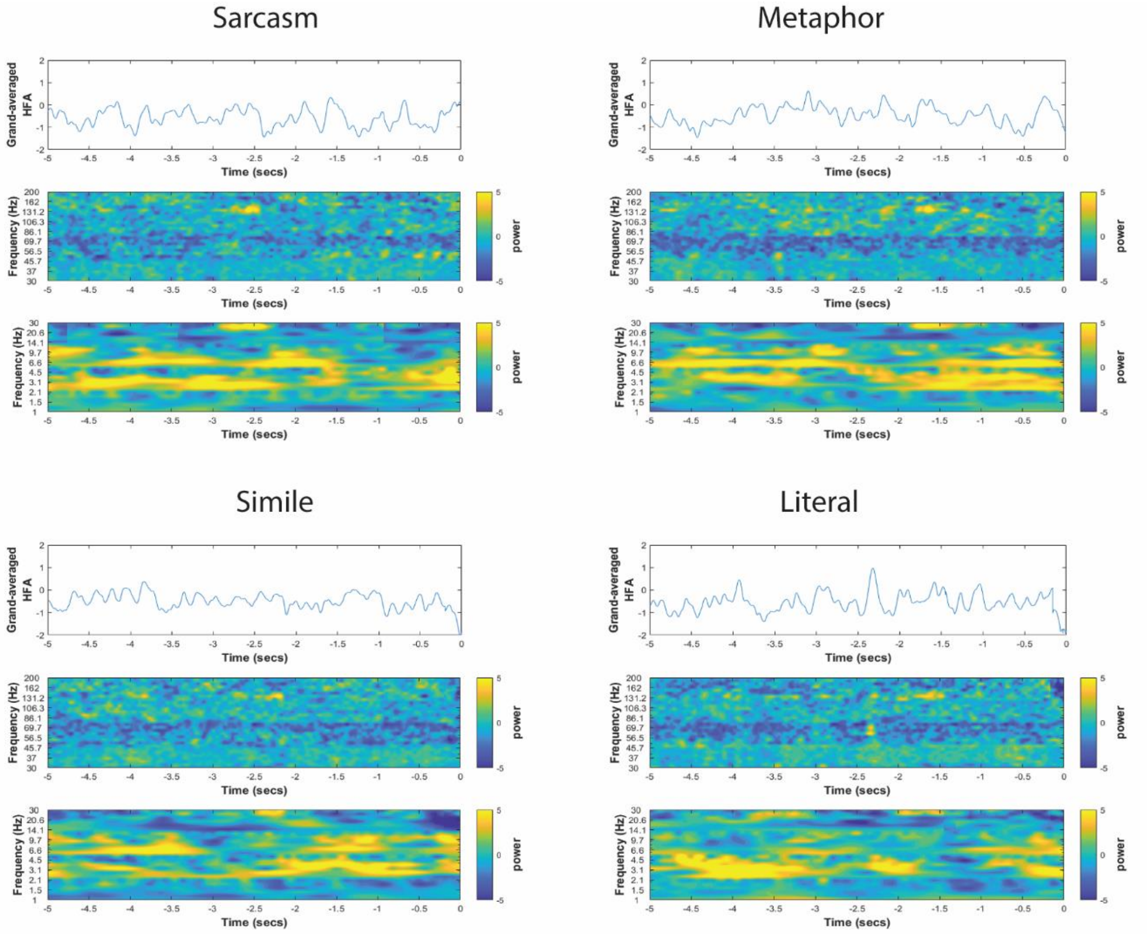

Supplementary Figure 2A. Grand-averaged high-frequency activity (HFA) and grand-averaged time-frequency representations of intracranial EEG activity at TPJ electrodes during response period. For the Response, t=0 indicates the start time of the participant's verbal response. For a given condition (sarcasm, metaphor, simile, and literal), spectral power was averaged within high-gamma frequency band (70-200 Hz) across all responses and then across all participants at TPJ electrodes to calculate grand-averaged HFA (*top* panel). X-axis and Y-axis represent time in seconds and power of HFA (in μV^2), respectively. For a given condition (sarcasm, metaphor, simile, and literal), time-frequency representations were averaged across all responses and TPJ electrodes and then across all participants, showing differential power modulations in both high (30-200 Hz, *middle* panel) and low (1-30 Hz, *bottom* panel) frequencies. X-axis and Y-axis represent time in seconds and frequency (in Hertz).

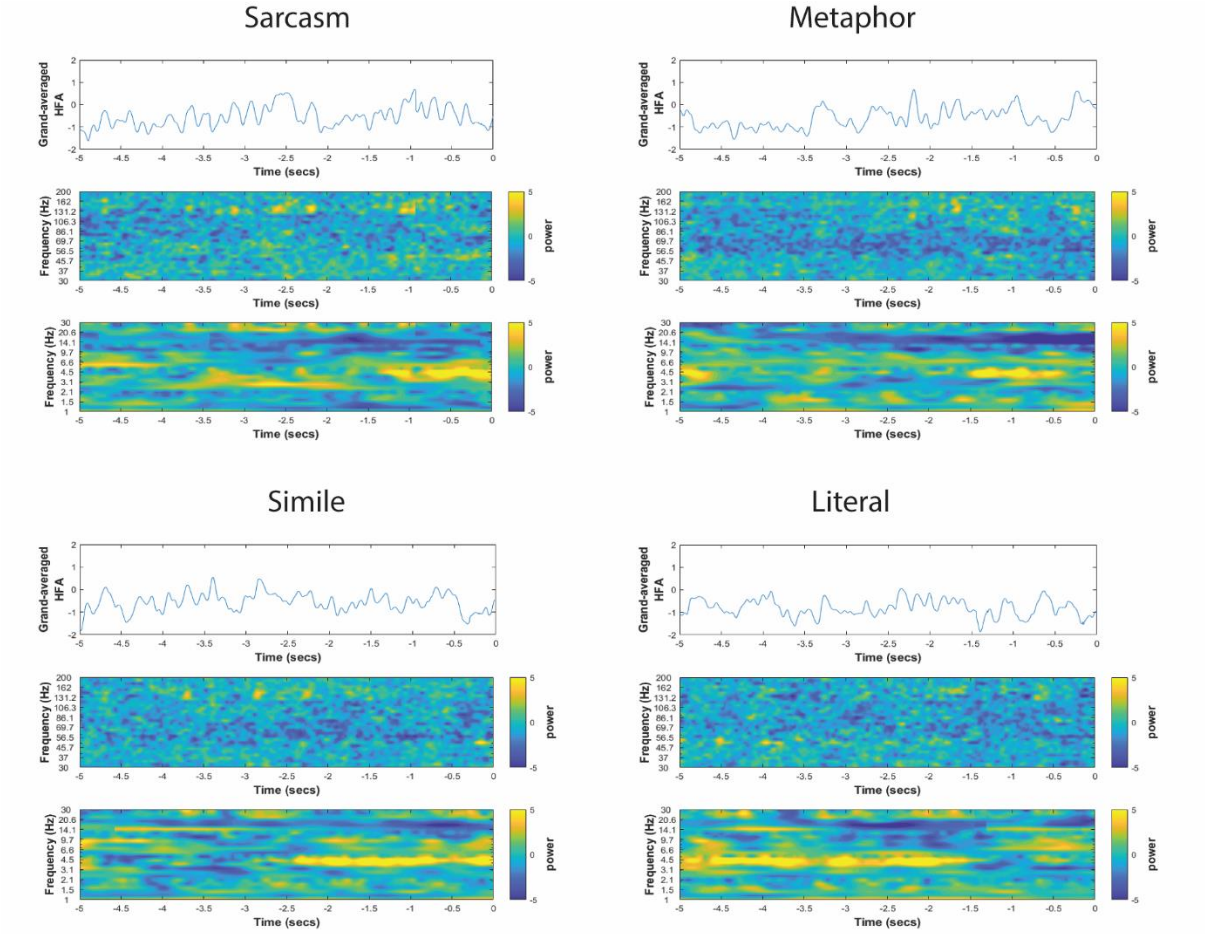

Supplementary Figure 2B. Grand-averaged high-frequency activity (HFA) and grand-averaged time-frequency representations of intracranial EEG activity at non-TPJ electrodes during response period. For the Response, t=0 indicates the start time of the participant's verbal response. For a given condition (sarcasm, metaphor, simile, and literal), spectral power was averaged within high-gamma frequency band (70-200 Hz) across all responses and then across all participants at non-TPJ electrodes to calculate grand-averaged HFA (*top* panel). X-axis and Y-axis represent time in seconds and power of HFA (in μV^2), respectively. For a given condition (sarcasm, metaphor, simile, and literal), time-frequency representations were averaged across all responses and non-TPJ electrodes and then across all participants, showing differential power modulations in both high (30-200 Hz, *middle* panel) and low (1-30 Hz, *bottom* panel) frequencies. X-axis and Y-axis represent time in seconds and frequency (in Hertz).

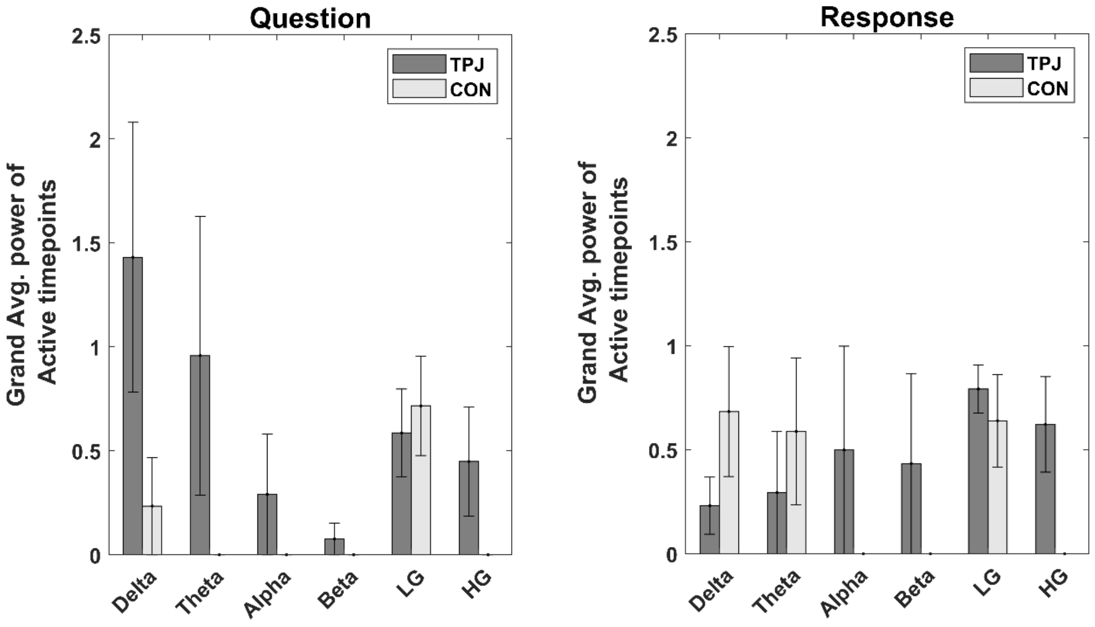

Supplementary Figure 3. Grand-averaged power of active timepoints (in $\mu V^2/Hz$) specific to non-literal processing for both TPJ and non-TPJ (CON = control) electrodes in all frequency bands averaged across participants. The power of significantly active clusters from non-literal versus literal contrast were averaged across all Questions/Responses, then across electrodes for both TPJ and non-TPJ control electrodes and then across all participants. The averaged powers were then compared in all frequency bands separately. Notably, the active clusters were averaged between -5 to 0 seconds for Questions and between 0 to 5 seconds for Responses. The comparison shows significantly more power related to non-literal processing at TPJ electrodes than non-TPJ electrodes in high-gamma ranges during Response periods. (two-sample t-test, $p=0.035$). There was no significant difference in power related to non-literal processing at TPJ electrodes than non-TPJ electrodes in any other frequency bands (two-sample t-test, $p>0.05$).
